# A Practical Guide to Avoiding Biased Communication in Reproductive Biology

**DOI:** 10.1093/icb/icae138

**Published:** 2024-08-13

**Authors:** Zoe Baker, Virginia Hayssen

**Affiliations:** Department of Biological Sciences, Smith College, 44 College Lane, Northampton, MA 01063, USA; Department of Biological Sciences, Smith College, 44 College Lane, Northampton, MA 01063, USA

## Abstract

When cultural biases pervade communication, whether visual or text-based, objectivity is impaired. Anthropocentrism (human-centered bias) and androcentrism (male-centered bias) in particular distort perspectives in mammalian reproductive biology. This paper provides a resource for professionals who understand how cultural biases can be reinforced with language, visuals, and conceptual framing. After brief explanations, we present neutral alternatives to biased terminology as well as ways to avoid bias in illustrations. Since this paper is animal-centric, we hope to inspire the creation of similar resources across a more diverse biota and, thus, move towards a more neutral perspective across reproductive biology.

As with other aspects of life, the words we use are, consciously or unconsciously, infused with our cultural heritage (Dwyer et al. 2022). The language of reproductive biology is not exempt from this cultural bias (Blackwell 1875; Kaminsky 2018; Hayssen 2020). Anthropocentric, androcentric, and value-laden terms both uphold and reinforce common misunderstandings and misrepresentations of reproductive processes, as can the graphics we use for illustration (Perry 1981; Beldecos et al. 1988; Martin 1991, 2001). For instance, in the early 1980s, Perry (1981) noted that the indiscriminate use of the word “egg” conflates three genetically different components of female reproduction: the diploid oocyte, the haploid female gamete, and the product of conception. This conflation leads to further misunderstandings, as, for instance, when discussing what an ovipositor releases: gametes (e.g., fish) or embryos (e.g., insects).

In the late 1980s, Beldecos et al. (1988) analyzed the importance of feminist critique in discussion of sex determination and conception, with an important example of how metaphors of violence and marriage influence the description of cellular processes. Subsequently, Martin in 1991 further detailed how culture shapes biological theories with a focus on the use stereotypical female–male roles in describing the function and actions of gametes. Then, a decade later, Martin’s (2001) book “The woman in the body” expanded her cultural analysis of reproduction by including an analysis of anatomical illustrations. As the work of these authors suggests, reducing bias in terminology and figures will allow for greater precision and accuracy in the presentation and understanding of reproductive processes. Beyond the specific utility of neutral language to describe form and function, these proposed alternatives are a small, but important, step towards reducing cultural bias, thus making science more objective.

Our intended audience is professionals who want to mitigate bias in their writing about reproductive biology, as well as individuals who may know the major issues regarding the types of bias, but perhaps not the specifics. Consequently, the paper is organized around several sources of bias: male-centered bias, eponyms, value-laden concepts with medical consequences, bias in illustrations, and global issues of bias. For each topic, we briefly define the issue and the concerns, give some examples, and provide alternative terms or frameworks, usually in the form of a table at the end of the section. Although the paper may be read linearly, it is structured such that individuals can focus specifically on the sections and additional resources with relevance to them.

When analyzing the harms of androcentrism and ways to mitigate perpetuating male-bias, we discuss another, related source of bias: anthropocentrism, human-centered bias. Although anthropocentrism is not the central bias explored in the paper, the “male” in androcentrism’s male-bias is implicitly a human male, who exists in dyadic hierarchy with a human female. Subsequently, the examples explored in the paper, though primarily mammal- and human-centric, incorporate critiques of anthropocentrism. Since we do not cover the rationale for every alternative, we provide additional resources at the end of sections when relevant. The work for this paper began at a SICB symposium in 2020 (Orr et al. 2020). Consequently, parts of this text borrow from Hayssen and Orr (2017, 2020); Hayssen (2020); and Orr et al. 2020.

The structure of analysis in Orr et al.’s (2020) round-table paper has inspired similar critiques of sexual and cultural bias in reproductive biology. Sharpe et al.’s (2023) SICB Symposium paper, drew inspiration from Orr et al. (2020) when organizing the work of “intersex activists and biologists working in a variety of systems across taxa who are critically engaging with language and concepts surrounding biological sex” (Sharpe et al. 2023, 960). As Sharpe et al. highlight: “Sex and gender are both complex and multifaceted [. . .] While some use the term “sex” in reference to one trait such as chromosomes, gonads, gamete production, or external genitalia, “sex” is often used to refer to many different traits with different distributions” (Sharpe et al. 2023, 961).

In this paper, we draw inspiration from Sharpe et al. (2023) in recognizing the “multi-faceted” nature of defining sex by making clear the specificity of the scope of our critique of androcentrism and our proposed ways of mitigating androcentrism. Throughout this paper, we use sex to refer to gamete production because our focus is on cultural bias in discussion of gametes (e.g., ovum versus “egg”). However, our definition is contextual to our analysis rather than exhaustive. For discussions regarding binarism and means of advocating for an intersex-inclusive perspective, we recommend the table of examples provided in Sharpe et al. (2023).

Some caveats: finding alternatives to biased language is challenging. Not all individuals will agree with our choices. Some individuals may find our alternatives euphemistic rather than meaningful (e.g., “intromittent organ” rather than “penis”). Some may find our alternatives meaningful for some taxa but not others. This is especially true as the authors come from a mammal-centric research orientation with concomitant bias. Even when an alternative term is meaningful, the practice of using the alternative term can remain challenging, particularly when a word is part of common speech, such as “egg” (alternative: ovum or zygote) or “fertilization” (alternative: syngamy or gamete fusion). When an alternative is completely novel (e.g., zygopositor for ovipositor), initial resistance can be expected. Change happens slowly; not all the changes we propose will be accepted. We hope that these challenges, and the thoughts that result, will prompt others to further the work from their own perspectives.

## Anthropo- and andro-centrism (human- and male-centered bias)

Human-centered bias stems from the cultural view that humans are the ultimate life form and, thus, superior to all others (Mylius 2018). Anthropocentrism is part of a philosophical tradition that can be traced as far back as Aristotle. The *Scala Naturae*, or great chain of being, is Aristotle’s hierarchy of life, wherein beings are organized according to proximity to perfection, which is synonymous with proximity to humankind (Mylius 2018). As a result, we may separate ourselves from other animals by giving different names to biological processes or anatomy in humans compared to analogous processes in non-human species. For instance, the preferential use of “Fallopian tube” rather than oviduct when describing human anatomy. Another side of anthropocentrism is the self-centered assumption that what is considered true for humans is also true for other life forms (e.g., emotional states). The *Scale Naturae’s* human ideal is a male human; the female perspective is absent in anthropocentric frameworks in that the non-male is outside of the hierarchy in the great chain of being. Thus, the female perspective is non-human-specific, although the examples and resources in this paper focus on mammals due to the authors’ area of research. Of course, even positing a female vs. a male perspective is also part of binary thinking.

The most obvious example of anthropocentrism in reproductive biology is the unjustified binary equivalency of sex and gender. Since at least Aristotle, the idea that all individuals, regardless of species, are either female or male has been part of the history of Western science (Sandford 2019). Further, the superiority of humankind within the framework of historical anthropocentrism is a male-specific, human superiority. This binary categorization of sex across taxa is often conflated with the concept of gender. However, gender refers to socially constructed roles or cultural norms; gender is a human attribute. Neither gender, nor sex, is binary. Unfortunately, “[w]ith our human perspectives, sex-specific predictions for females and males may be unconsciously influenced by culturally specific gender-biased assumptions” for other taxa (Ahnesjö et al. 2020). Extreme examples include using human-centered terms for plants (e.g., placenta, Henry et al. 2020; gender, Vyskot and Hobza 2004), fungi (e.g., yeast sexes and the conflation with mating types, Lachance et al. 2024), and bacteria (e.g., sex, Bivins 2000).

In mammalogy, a less obvious example of anthropocentrism is equating the human reproductive cycle, as observed in so-called Western countries, to the general mammalian reproductive cycle (Hayssen and Orr 2017, 73). As Conaway (1971, 239) stated over 50 years ago, “[i]n natural populations the nonpregnant cycle is a rarity, and it is essentially a pathological luxury which cannot be tolerated.” With contraception, human females (and captive mammals) can undergo repeated hormonal cycles without reproduction. For humans [and a few other mammals, e.g., tree shrews, *Tupaia* (Conaway and Sorenson 1966)], this cycle is from menstruation to menstruation.^1^ However, most mammals absorb the uterine endometrium when conception does not occur. For these mammals, the repeated cycle is from ovulation to ovulation, which is sometimes accompanied with a visible behavioral cue called estrus. Such repeated cycles are called estrous cycles. However, in natural populations, continuous estrous cycling is aberrant. The usual reproductive cycle for adult female mammals is ovulate, conceive, gestate, lactate, and then repeat the process or shut down the system when conditions support energy conservation rather than reproduction (e.g., drought, winter) (Hayssen and Orr 2017, 100). In contrast, domesticated, laboratory, or zoo animals usually have unrestricted resources and often protection from environmental stresses (e.g., predators, parasites, weather) (Hayssen and Orr 2017). These individuals have the energy for reproduction but their offspring production is under human control. Thus, these repeated estrous cycles are an artifact of captivity and the concept of an estrous (or menstrual) cycle is of human design.^2^

The repeated estrous cycling is often treated as the usual condition for mammals in the wild. It is not. Continuous ovulatory or menstrual cycles without offspring production are a byproduct of captivity or other abnormal human-derived conditions (e.g., domestication). Unfortunately, much reproductive science is based on assessing components of the estrous cycle (e.g., the luteal phase), components, which are not a significant part of reproduction in natural populations (Hayssen and Orr 2017). Using these human-designed concepts may hinder conservation efforts to either increase or decrease population size.

Social roles associated with humans and binary thinking also creep into reproductive language. As a short example, the cultural understanding of testosterone is especially mismatched with its biology (Jordan-Young and Karkazis 2019). Testosterone is identified as a “male” hormone with links to “male” qualities (e.g., aggressiveness, etc.), when in fact, many of these neural effects are because testosterone (an androgen) is aromatized to estradiol (an estrogen) (Adkins-Regan 1990). Thus, a “female” hormone, estradiol, is associated with male behavior. Conversely, testosterone is positively associated with partner cuddling and reactions to crying babies, which are actions culturally associated with human-female behavior (Bos et al. 2010; van Anders et al. 2011). Furthermore, questioning cultural perceptions of testosterone as a primarily male hormone allows for investigation into testosterone’s role as a female hormone. For example, the chapter “Ovulation” in “Testosterone: an unauthorized biography” questions the exclusion of femaleness from common definitions of androgens: “[A]ndrogens are [. . .] the hormones that generate ‘maleness,’ and the lingering concept of sex hormones suggests this will get in the way of ‘femaleness’ ” (Jordan-Young and Karkazis 2019, 43). Jordan-Young and Karkazis conclude the chapter by proposing that testosterone and its fellow androgens “playing a central role in ovulation undermines their very classification as androgens” (Jordan-Young and Karkazis 2019, 61). Here, androcentrism contributes to research gaps, in precluding the possibility of “male” hormones playing roles in “female” processes.

As the above examples suggest, equating hormonal effects with cultural roles is misleading. Thus, defining an androgen as “a masculinizing substance” (Barresi and Gilbert 2024) restricts the multiple actions of the molecule to a binary classification. In addition, giving human-centered names to substances complicates research when researching the same molecules in other taxa (Fodor et al. 2020). This is especially problematic in genomics and transcriptomics, where “homologous genes found in different species are presumed to perform homologous functions” (Fodor et al. 2020, 1). As Elizabeth Adkins-Regan put it: “The association of androgens with masculine traits and estrogens with feminine traits is also a poor fit with nature’s ways” (Adkins-Regan 2005, 6). The terms feminize and masculinize not only have a binary bias, but also a vertebrate-centric one. If used as replacements, “estrogenize” and “androgenize” have the same problems. Until more neutral terms are found, we suggest describing the specific changes in anatomy, behavior, or physiology rather than relying on a single, more general term.

Of course, one major social construct is the binary sex-categorization designating individuals as either “female” or “male.” In this paper, we, simplistically, use the words “female” and “male” to refer to gamete type in those animals that have dichotomous, haploid gametes (usually of different sizes). However, as Smiley et al. (2024), remark “sex is observable across many levels of biological organization, including genetic, molecular, cellular, physiological, behavioral, social, and ecological levels, which may or may not be congruent” (Smiley et al. 2024, 105445). Smiley et al. (2024) also provide clear definitions (their Tables 1 and 2) of terms associated with sex diversity and variability. Recognition of the diversity of “sex” is a necessary first step towards reducing bias in research and combatting anthropo-androcentrism.

Overall, social stereotypes obscure the reproductive biology that we are trying to objectively understand, study, and teach. Avoiding anthropocentric terminology helps reduce the influence of hidden assumptions (Table 1). Neutral terminology and phrasing will also help us to examine unexpected results with an open mind and allow us to see such results as interesting variations we had not previously considered. In other words, treating the unexpected as opportunities to explore, not exceptions to explain away (Ahnesjö et al. 2020).

**Table 1:** Options to reduce anthropocentric bias. Often avoiding a term with a broad binary generalization is the best option.

Human bias	Alternative
Feminization, feminized	Avoid; describe the anatomy, physiology, or behavior
Heterosexual	Different-sex behavior (not “opposite” sex)
Homosexual	Same-sex behavior
Masculinization, masculinized	Avoid; describe the anatomy, physiology, or behavior
Penis	Intromittent organ
Sex roles	Avoid; describe the anatomy, physiology, or behavior

**Fig. 1:**
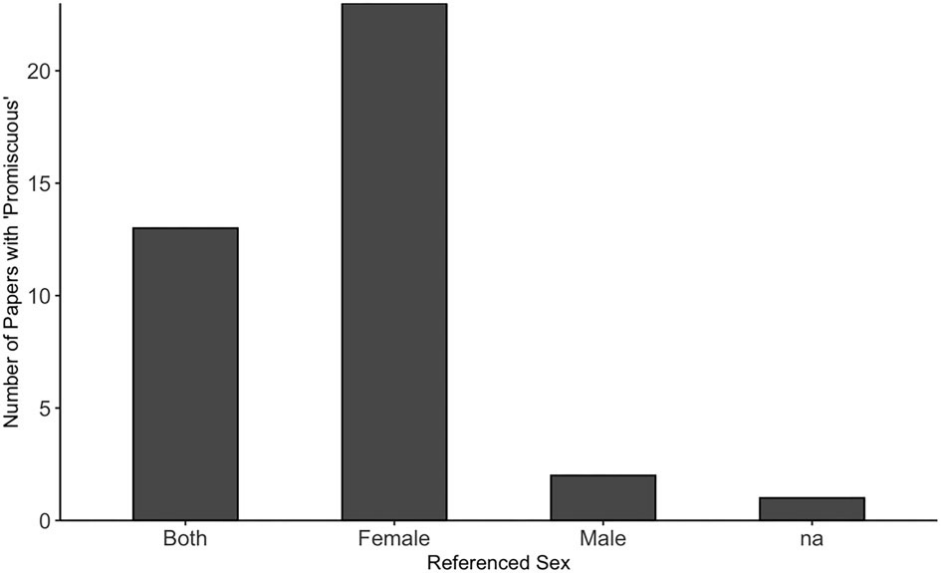
Data from Elgar et al. (2013). Analysis of 39 papers published in the journal “Animal Behaviour” between 2000 and 2010 on the association of “female” or “male” with the keyword “promiscuous” (and its associated derivations) in either the abstract or main text.

Penis vs. Intromittent organ: We include “penis” in this section since the reproductive biology of a Brazilian cave insect challenges the human concept of a penis (Yoshizawa et al. 2018). The term “penis” has both anatomical and functional meanings. Anatomically, a penis is part of a male reproductive system,^3^ whereas, functionally, a penis is a sperm-transfer organ. The genus *Neotrogla* (order Psocodea) is a tiny Brazilian cave insect in which sperm transport is via female, not male, structures. Specifically, “females have a penis-like intromittent organ. . . which is inserted into a male vagina-like genital cavity for copulation” (Yoshizawa et al. 2018, 2; Fig. 1A). Copulations are 40–70 h and spines on the female penis “anchor a male coercively during copulations” (Yoshizawa et al. 2018, 2). Females use the semen for both reproduction and nutrition. Females have large storage organs (spermatheca) and a switching valve, which allows them to receive a second seminal packet (from the same or a different male) while the first is consumed. Thus, female *Neotrogla* achieve intromission and sperm transfer with their gynosome. One could reasonably ask, why not add “vagina” to the table. Here, the answer is etymological, as the origin of the word “vagina” is from Latin for “sheath” or “scabbard” (Hayssen 2020), in other words, a receptacle for an intromittent organ.

**Fig. 2:**
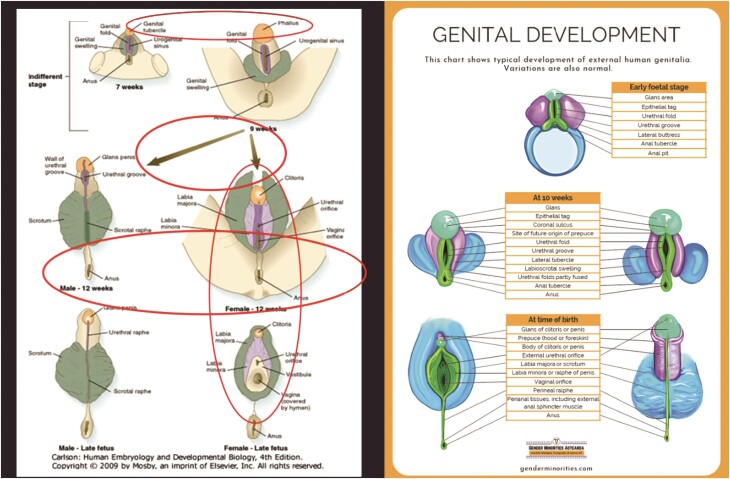
Two graphics illustrating human genital development. Left: development of males and females from 7 to 12 weeks and before birth from a 2009 developmental biology textbook (modified from Fig. 16.41, Carlson 2024, 413). Ovals encircle elements of bias as detailed in the text. Right: development of females and males over a similar developmental period from a 2023 website devoted to transgender public health in New Zealand (Gender Minorities Aotearoa, genderminorities.com).

Even without *Neotrogla*, intromittent organ may be preferable. Although the penis of amniotes is homologous, it is also homologous with the clitoris, since external genitalia have the same embryonic origin (Sanger et al. 2015 and Fig. 2 below). However, not all animals use a penis to transport sperm during mating. For example, sharks use claspers, which are modifications of pectoral fins. Sperm transfer via an intromittent organ is accomplished by modifying a variety of body parts such as sensory organs (spiders), limbs (insects), and tentacles (squid) (Brennen 2016). Thus, use of “intromittent organ” allows the term to maintain the functional use without the androcentric or amniote bias.

## Resources to reduce anthropocentrism

Our example of the mismatch between the cultural and biological understanding of hormones is thoroughly explored in Jordan-Young and Karkazis’s (2019) book “Testosterone: an unauthorized biography.” This book explores the truths and myths regarding what testosterone does across six domains: ovulation, violence, power, risk-taking, parenting, and athleticism. In doing so, the narrative makes transparent the effects of social context on the process and progress of science.

Specific to binary-gender bias, Ahnesjö et al. (2020) published excellent guidelines for awareness of gender-biased assumptions as well as recommendations for study designs and terminology to reduce the unintended consequences of cultural biases. They also remind us that sex, sexuality, and gender are not synonymous.

Similarly, Massa et al. (2023) provide guidelines for experimental design and methodology. They note, for instance, that “sex” is not a mechanism, a biological variable, or a dimorphic trait, but is, instead, a category constructed within a cultural system. Their paper provides specific questions to ask before one conducts research or analyzes one’s results. While their work focuses on neuroendocrinology and behavior, the questions themselves have broader relevance.

The historical use of gendered language in bacterial genetics was thoroughly explored by Bivins (2000) over 20 years ago, and the topic is ripe for continued research. More generally, Sandford (2019) examines the history of “sex” as a natural category.

## Ambiguity and misunderstanding as a result of androcentrism

The origins in antiquity of androcentrism mirror those of anthropocentrism. The anthropocentric logic of *Scala Naturae* evaluates all non-human life by how similar non-human species are to humans, whereas the androcentric logic of Plato’s ideal form, which is a male, evaluates the female based on her similarity to the male ideal (Hibbs 2014). Hence, concepts and terminology from the male-biased point of view may identify insufficiency (fewer gametes) where there is only difference. They may fail to account for the realities of female reproduction that challenge androcentric frameworks (e.g., egg vs. sperm, solicitation vs. receptivity).

One critical tenet of androcentric thought is that females are passive subjects and males are active agents. Current reproductive biology continues this outdated narrative (e.g., the “sperm race”). For example, research articles about reproductive behavior as well as gynecology textbooks selectively use words related to passive action in descriptions of females and active words to describe males (Bertotti Metoyer and Rust 2011; Green and Madjidian 2011). These cultural stereotypes prevent both students and scientists from progressing to an objective understanding of reproductive biology (Hayssen 2020). Here are examples of specific words and phrasing to avoid ambiguity and misunderstanding.

Ovum/Ova or zygote (not egg). “In laboratory parlance, and even in print, the oocyte [. . .], ovum, zygote, morula, and blastocyst are frequently referred to indiscriminately as the ‘egg’ ” (Perry 1981, 321). The inaccurate, imprecise language of “egg” conflates the female gamete (an ovum) with the product of conception (a zygote) (Hayssen and Orr 2017). Female gametes (ova) are haploid, single cells that do not divide, whereas zygotes are diploid fusions of female and male genetic material that subsequently divide repeatedly to produce individual organisms.^4^

Haploid gametes have short lives, whose physiology is mostly regulated by their diploid parent (Krisher 2013). They have limited nuclear gene expression. In contrast, after the first cell divisions, expression of nuclear DNA of a zygote regulates most of its physiology and development. Evolutionarily, ova compete with other ova for sperm, whereas sperm are not an evolutionary resource for zygotes, although parental investment may be. Thus, ova and zygotes are not the same, anatomically, physiologically, embryologically, or evolutionarily.

The use of “egg” for both the female gamete and the product of syngamy occurs even in a 2021 review of invertebrate oogenesis. In this paper, Eckelbarger and Hodgson (2021) define oogenesis as the “process of converting oocytes into eggs,” thus equating female gametes with “eggs” (Eckelbarger and Hodgson 2021, 2). As unintended justification, they note that “eggs” fascinated Aristotle (4th century B.C.) and William Harvey (1578–1657). But, since knowledge of female gametes was unknown until much later, the eggs that fascinated Aristotle and Harvey are embryos, not female gametes. Thus, in this very technical paper, the authors use the word “egg” for both the product of syngamy and the female gamete.

Unfortunately, the conflation of zygote and female gamete makes etymological sense. In fact, since “ovum” and “ova” come from the ancient Greek word for “egg,” English (and romance languages) do not have a unique word for female gametes. Unless we devise a new word for female gametes, the best course of action seems to be to use “ovum/ova” and refrain from using the word “egg” as a synonym.

Not surprisingly, the phrase “unfertilized egg” is also problematic. When syngamy occurs, the result is a diploid zygote, not a fertilized ovum. Also, when syngamy does not occur, an ovum is still an ovum, not an unfertilized ovum. Oocytes are the diploid precursors to haploid ova. However, as noted above, oocytes are also called “eggs.” The confusion using “unfertilized egg” specifically arises when defining parthenogenesis. For instance, in a review of invertebrate reproductive modes, Subramoniam (2018, 36) defines parthenogenesis as “the development of a new offspring from an unfertilized egg.” Then they define meiotic parthenogenesis “as the fusion of the egg with the second polar body,” thus directly equating an “egg” with the haploid female gamete (Subramoniam (2018, 36). However, when explaining that apomictic parthenogenesis “entails modification or absence of meiosis so that the eggs remain diploid,” the author expands the definition of “egg” to include the precursor cell, the oocyte. Thus, in one paragraph, the author uses the word “egg” in very different biological capacities, succinctly illustrating the problematic nature of the phrase “unfertilized egg.”

Ambiguity: Ovipositor and Oviparous Another consequence of not having a distinct word for female gametes is that terms derived from “ovum” or “ova,” such as oviparity and ovipositor, maintain the ambiguity. Biologists (Wourms and Lombardi 1992; Blackburn 2015) studying viviparity have dealt with the ambiguity of oviparity by substituting separate terms for the release of female gametes, zygotes, or embryos from a female’s reproductive tract: ovuliparity (ovuliparous), zygoparity (zygoparous), or embryoparity (embryoparous). These terms have been accepted and are currently in use (Fukakusa et al. 2020; Ringvold and Vesterinen 2021; Kotenko and Ostrovsky 2023). The ambiguity of “ovipositor,” however, has not received attention.

An ovipositor is considered an “egg-laying” structure. But what does an ovipositor deposit: gametes or embryos? In fact, depending on the taxon, either can be released. For many insects with internal syngamy, females release zygotes (or embryos) via their ovipositors. In contrast, for fish with external syngamy, the female’s ovipositor usually deposits female gametes (ova). For instance, females of the parasitic bitterling (*Rhodeus ocellatus*) deposit their ova in the siphon of their host, a freshwater mollusk, after which, males spawn into the same siphon (Casalini et al. 2013; Dykova and Reichard 2023). Uniquely, syngnathid females (seahorses and pipefish) use their ovipositor to deposit ova into a male’s pouch, where “internal” syngamy occurs (Holt et al. 2022; Schneider et al. 2023). Surprisingly, in several groups of bony fish, females oviposit both ova and sperm simultaneously before syngamy but after copulation (cottids: Petersen et al. 2005; sculpins: Awata et al. 2019), the result of a process called “internal gametic association.” In all these cases, females with ovipositors are said to lay eggs. Clearly, to use the word “egg” for ova, sperm, and zygotes is not only imprecise but can easily lead to misunderstanding the basic reproductive biology that occurs. We suggest that “ovipositor” be used only for the deposition of female gametes, and that zygopositor^5^ be used for deposition of zygotes or early embryos.

Removing the word “egg” from the commonly used English language would be impossible and unnecessary. That said, in scientific and educational communications, we should be able to unambiguously refer to female gametes or zygotes, rather than calling them both “eggs.”

Conception, gamete fusion, or syngamy (not fertilization, impregnation). The terms fertilization and impregnation are female-passive/male-active, whereas “conception,” “gamete fusion,” or “syngamy” are gender-neutral alternatives. However, to establish the regular use of these neutral alternatives, we need to be comfortable with using gender-neutral phrases, such as internal conception, external syngamy, delayed gamete fusion, or artificial reproduction. These phrases may seem awkward to use now, but that is because they are not familiar yet. Even so, familiar acronyms have simple equivalents. For artificial reproduction, IVF could refer to “*in-vitro* fusion” rather than “*in-vitro* fertilization.” For embryology, DPC could refer to days past conception rather than days past copulation, a change that would more accurately refer to the age of the embryo.

Androcentric phrasing to avoid. Phrasing and use of common words can maintain bias and reinforce hidden assumptions. Here are some specific things to avoid.

*Gendered verbs*. As a corollary to the comments on conception, “fertilize” is a verb with no female-centric or neutral alternative, that is, to conceive of an idea is not the same as to fertilize it. The same issues are evident with “impregnate.” Similarly, in conception, does the ovum engulf a sperm or does a sperm penetrate an ovum? Rather than either metaphor, try “the gametes fuse” or “ovum and sperm fuse.”

English has other gendered verbs that stem from traditional cultural stereotypes and carry cultural overtones. For instance, to father an offspring is the same as to sire one, but to mother an offspring is not the same as conceiving one.

*Concepts that have a cultural bias*. For instance, “virile” describes sexual strength and energy, which are positive traits in females and males, but its synonyms are manly, masculine, or male. English has no word for female sexual strength and vitality.

The juxtaposition of “male promiscuity and female adultery” in a 1983 article about monogamous rooks (Røskaft 1983) would be flagged as inappropriate today, but a 2023 article about “divorce rate in monogamous birds” (Chen et al. 2023) indicates that cultural language continues to invade objective scientific studies.

The word “promiscuity” has additional concerns. “Promiscuity” is commonly used for females that mate with more than one male but much less used for males that mate with more than one female (see Fig. 1; Elgar et al. 2013). Further, Elgar et al. (2013) identify the vagueness of terms such as “promiscuity”: “Promiscuous has been used as an umbrella term to include polyandry, polygyny, and polygynandry” (Elgar et al. 2013). As evidence, the authors examined 39 papers from the journal *Animal Behavior* between 2000 and 2010 that included the term “promiscuous” (and its derivatives) (Elgar et al. 2013). They recorded to which sex the term “promiscuous” (or its derivatives) referred, as well as whether authors inferred (or suggested) “pre-copulatory female choice” in a species (*n* = 18) (Elgar et al. 2013). Not only was “promiscuous” used more commonly for females (Fig. 1), but “promiscuous” was often used ambiguously to describe myriad sexual behaviors: “pre-copulatory female sexual selection” described, *n* = 18; “no female sexual selection described,” *n* = 16; and “not applicable,” *n* = 5 (Elgar et al. 2013). Thus, not only does usage of the term “promiscuous” potentially introduce cultural biases, but also the term is imprecise regarding what sexual behaviors it includes.

The order of words unintentionally conveys priority. Here are some examples primarily from phrasing associated with humans: “males and females,” “husband and wife” (or “man and wife”), “ladies and gentlemen.” All these pairings, and others, come from binary thinking and usually have a common order of precedence in English parlance. The pairings should be used thoughtfully.

*Avoid metaphors*. Martin (1991) explores how cultural stereotypes pervade descriptions biologists use to describe reproductive biology. For example, an article on conception described antrum formation as “the ripening follicle,” as though the follicle were a fruit to pluck and eat (Bedford et al. 2004, 894); the same article did not use metaphors when describing gamete maturation in males. As with “adultery” above, “cuckold,” “coy,” and “divorce” (Milam 2012; Laczi et al. 2021; Chen et al. 2023; MacGillavry et al. 2023) are terms still in use that equate human cultural stereotypes with animal behavior.

Verbs also convey metaphorical action. For instance, does a female “exploit” an ejaculate or a spermatophore or is a male “manipulating” a female with his “gift.” Similarly, “sperm competition” and “sperm race” have cultural connotations that may not match the biological reality (Hayssen 2020).

*Avoid misleading definitions* that introduce fallacies and force male bias. For example, females become sexually mature “upon first ovulation,” not when they are “capable of being fertilized” (Boness et al. 2002). Refrain from describing female behavior from a male perspective; instead, use “pro-copulatory behavior,” not “receptivity,” and use “solicitation,” rather than “attractivity” (Hayssen 2020).

Importantly, androcentrism (Table 2) is, in part, a result of binary thinking. In fact, much of reproductive biology uses the binary, female vs. male categorization. Increased awareness of multiple forms of reproduction may make androcentrism obsolete.

**Table 2:** Alternatives to ambiguous or androcentric terms. As with all these tables, the terms are not perfect and we invite others to explore different options.

Androcentric term/Concept	Alternative
Artificial insemination (AI)	Assisted reproduction (AR)
Attractivity	Solicitation
Egg (female gamete)	Ova/ovum
Egg (“fertilized egg”)	Zygote, blastocyst, conceptus, and embryo
Female phallus, female penis	Clitoris or Enlarged clitoris*
Fertilization (delayed, external, *in vitro*, assisted, etc.)	Syngamy, gamete fusion, conception (delayed, external, *in vitro*, assisted, etc.)
Induced ovulation	Facultative ovulation
Oviparity (oviparous)	Zygoparity (zygoparous) and embryoparity (embryoparous)
Ovipositor	Zygopositor
Primordial phallus	Genital tubercle
Receptivity	Pro-copulatory behavior

*The neutral alternative of “enlarged clitoris” here is proposed for non-human animals. The table is a resource for combatting anthropocentric androcentrism, and the language used for human anatomy can vary in the context of gender.

## Resources to reduce androcentrism

Wasser and Waterhouse (1983) compiled male-oriented explanations for the following concepts: reproductive synchrony, continuous receptivity, concealed ovulation, and orgasm in women.

Donna Haraway’s (1989) “Primate Visions: Gender, Race, and Nature in the World of Modern Science” critically exposed how the academic and popular understanding of primate behavior is shaped by western narratives and metaphors.

Hrdy (2000) explores androcentric bias in descriptions of sex drive and libido.

Milam (2012) examines the history and use of “coy” as either an active or passive component of animal behavior and sexual selection.

Hayssen (2020) details misconceptions about conception and discusses other types of bias in anatomical terminology.

## Eponyms

An eponym is a name given to something (for instance in biology, a disease, anatomical structure, or species) that is derived from a real or imaginary person. In practice, eponyms are a combination of both anthropocentric and androcentric thinking. By deriving the name for an anatomical structure from an individual, the act of “discovery” is privileged over communicating a function or describing the anatomical structure. By connecting an individual (usually a White, Western man) with an anatomical structure and, sometimes with implied ownership (e.g., Skene’s gland), eponyms directly introduce subjective and cultural bias into science (McNulty et al. 2021).

Eponyms may highlight individuals who provided initial descriptions of anatomical structures in medical literature. Yet when primacy of description is unclear or contested, eponyms cannot communicate the very information that they are intended to communicate. For example, the eponym “Skene’s gland” recognizes the contributions of Alexander Skene in describing the paraurethral glands and ducts (see the authors’ discussion in the section “The female prostate: revisited” below). However, Alexander Skene’s contributions were published in 1880, which is over 200 years after Regnier de Graaf first described the tissue in 1672 (Biancardi et al. 2017). Further, for some, eponyms are, by their nature, offensive: “The truth is, men are all over women’s bodies—dead, white male anatomists, that is. Their names live on eponymously, immortalized like audacious explorers for conquering the geography of the female pelvis as if it were *Terra nullius*” (Kaminsky 2018).

In fact, several parts of female reproductive anatomy, from Graafian follicles and Fallopian tubes to the G-spot, have been named after men, but no male reproductive anatomical structures are named after women (Hayssen 2020). Using male eponyms for female anatomy focuses on the “historical victories of men ‘discovering’ body parts” (Kaminsky 2018). The subliminal message is that female body parts are objects that are important for the male who “discovered” these anatomical structures rather than their reproductive function (Kaminsky 2018). But the reproductive function of reproductive anatomy is what matters to scientists, and since we have alternative names that focus on function, rather than discovery,^6^ we should use those (Table 3).

**Table 3:** Eponyms with alternates that focus on anatomy or function.

Eponym	Alternative
Bartholin’s Glands	Greater vestibular gland; bulbourethral gland
Fallopian tube(s)	Oviduct(s)
G-Spot (Grafenberg spot)	Erogenous zone, erogenous spot
Graffian follicle	Preovulatory or mature follicle
Pouch of Douglas	Rectouterine cul-du sac, rectouterine pouch, rectovaginal pouch when uterus or vagina is present*
Skene’s gland	Prostate or paraurethral gland, if the tissue is not considered prostatic [not female prostate]

*A small extension of the peritoneal cavity near the reproductive system, called the retrovesical pouch when seminal vesicles are present.

Indeed, the Federative Committee on Anatomical Terminology (a committee of the International Federation of Associations of Anatomists) provided the following statement at the time of publishing *Terminologia Anatomica:* “The committee has continued the previous standard in not using eponyms. Despite their historical interest, honoring those who had first described, drawn attention to, demonstrated the meaning of, or correctly interpreted a particular structure, eponyms give absolutely no anatomical information about the named structure, and vary considerably between countries and cultures” (Whitmore 1999, 51).

## Resources for eponyms

For more on eponyms in taxonomy, see Guedes et al. (2023), who argue that eponyms have no place in 21st-century biological nomenclature. Or, see Nicholas Lund’s remarks on eponyms and ornithology in the article, “Dropping Names”: “Why [are we] stuck with names decided on a whim hundreds of years ago, especially when the names aren’t very good” (Lund 2024).

## Medical consequences and value-laden terms

Anthropocentrism and androcentrism have implications for medical outcomes and conservation efforts. Value-laden terms are another vector for bias in medical contexts. For instance, the implication of insufficiency or error on the part of a female may bias legislation or may even cause a miscarriage of justice (pun intended). The distorting effects of bias lead to the proliferation of inaccurate analyses and widening research gaps. Table 4 is a list of value-laden concepts paired with relatively neutral alternatives. Preceding the table are examples of the impact of value-laden terminology on identifying research gaps and developing methodologies. But first, we use the prostate as a case study of androcentrism’s impact on research in female reproductive biology.

## The female prostate: revisited

“…*naturally, the differences in organ parameters between males and females should not be an adequate argument to support the simplistic view that some organs in women are inferior to those of men. A similar conclusion also applies to the female prostate: it cannot be considered inferior just because it is smaller and has a smaller weight than the male prostate*…” (Zaviačič 1999 as cited in Biancardi et al. 2017).

The prostate and its function in male mammals have been studied since 335 B.C.E., whereas the prostate in females, though a homolog of the male prostate, remains understudied and often is referred to with misleading language (Tomalty et al. 2022). Using ultrastructural observations of the secretory epithelial cells and histological analyses of adult human prostate glands in females, the homology of paraurethral glands as initially identified in de Graaf’s writings is now known (Zaviačič 1999, as cited in Biancardi et al. 2017).

Though research has stressed the homology of the prostate across sexes, the putative biological functions of the prostate in females remain under-researched (Zaviačič 1999). Further, though diseases of the prostate in females are more rare than those in males, prostates across sexes are susceptible to lesions (Biancardi et al. 2017). By using the more accurate term of “prostate” to refer to the paraurethral glands and ducts rather than “Skene’s gland,” the homology of the prostate across sexes is emphasized, which may help call attention to research gaps regarding diseases and function of the prostate in females. Particularly, the use of the term “prostate” may remind practitioners that cancer of the prostate can occur across sexes, although most common in cisgender males (Thum et al. 2017; Dell'Atti and Galosi 2018; Muermann and Wassersug 2022; Chen et al. 2024). For instance, Slopnick et al. (2022) identified^7^ 15 cases of “adenocarcinoma resembling prostate” within 211 articles published 1974–2022. For the 15 cases identified, the median age of the female patients was 71 years old (Slopnick et al. 2022). Additionally, see a report of three cases in Singh et al. (2017) for discussions on the dearth of research on the effects of long-term testosterone hormone replacement therapy on the genitourinary tract of transgender men (FTM). Singh et al. (2017) present histological findings of “mesonephric remnants show[ing] epididymal differentiation and prostate-type glands within the cervical squamous epithelium of FTM transgender[. . . men]” (Singh et al. 2017, 333). Understanding the equivalence of prostatic tissue across sexes will become more and more important as access to medical care for trans individuals becomes increasingly available.

## The impact of value-laden concepts

Short luteal phase vs. luteal deficit (or defect): In human gynecology, a luteal deficit is condition where the endometrium (uterine lining) is not thick enough for implantation and a subsequent pregnancy would be disrupted. Even if measuring the thickness of the endometrium were simple, when to make that assessment is not clear. Thus, gynecologists use the length of the luteal phase, or the amount of progesterone in the blood, as a substitute and women with shorter luteal phases or lower progesterone levels are said to have a luteal deficit. However, data on endometrial thickness, progesterone levels, and the length of the luteal phase in rural Polish women compared to urban US women of similar age (27–28 years) had lower progesterone levels as well as a shorter luteal phase, but these differences did not lower fertility. In fact, lower hormone levels were associated with higher fertility, as 73% of the rural women had children compared with none from the urban sample (Clancy et al. 2009). These complicated results challenge one medical practice in fertility regimes, which is to administer hormones (chiefly progesterone) at higher than physiological levels to lengthen the luteal phase.

Miscarriage: In humans, embryo rejection is common before implantation. Chemical communication between the embryo and the female is necessary for pregnancy. Unless the conceptus signals its presence, it will be sloughed off. In fact, in humans, many early pregnancies are naturally lost before 13 weeks (9–17% of recognized pregnancies in women 20–30 years and up to 75–80% for women at 45); the cause of gestational loss is usually (∼60%) fetal chromosomal abnormalities (ACOG: American College of Obstetrics and Gynecology 2018). Thus, embryo rejection is not a mistake. It is common and necessary. Although “miscarriage” is one of the few terms in Table 4 that give females agency, that agency is used to imply that gestational loss, *sensu lato*, is due to the female making an error for which she is consequently to blame.

**Table 4:** Value-laden concepts paired with relatively neutral alternatives.

Value-laden term	Alternate
Blighted ovum	Anembryonic gestation
Cervical incompetence, incompetent cervix, and cervical insufficiency	Early cervical dilation, cervical funneling, and short cervical length
Cervical ripening	Cervical effacement
Luteal deficit (defect)	Short luteal phase
Miscarriage	Spontaneous abortion, embryo rejection gestational loss, and pregnancy loss

## Bias: not just in text

*“[D]espite increased attention to gender issues in medicine, the visual representation of gender in medical curricula continues to be biased”* (Parker et al. 2017).

Figures and tables have always been components of printed scientific textbooks and research articles. Historically, images, especially in color, were expensive to print, so the number of figures was constrained. That constraint is much less with digital media, and, currently, digital media are the major outlet for research, textbooks, and educational websites. As a result, graphics have proliferated, and the potential for visual bias has also proliferated.

One of the most effective ways to avoid bias is to actively look for it. In other words, to be aware of the potential sources of hidden sources of bias and find them. Bias in graphics can be cultural but can also be related to either design or content.

One major source of content bias is because images are 2-D and static, whereas the phenomena being illustrated may be 3-D and dynamic. One common example is depicting the development of a female germ cell from the earliest stages within primordial follicles through ovulation, corpus luteum formation, and atresia. Many illustrations depict all the stages in a cycle around the periphery of an ovary. This depiction conflates temporal change with ovarian location. But developing follicles do not travel a path around the ovary. They get jostled; they get larger or smaller (expand/contract); and they push other follicles out of the way or get squished themselves, but they do not parade in an orderly fashion around the ovary.

A second content issue is when a static image implies forceful movement. An example is illustrating ovulation as though it were a rapid volcanic eruption with an ovum bursting forth as it is expelled. Even these verbs (burst and expel) imply fast action, although ovulation may well be slow and tempered. In fact, ovulations induced from exteriorized ovaries of anesthetized rabbits lasted ∼10 min for extrusion, not counting the prior 88 min in which blood left the eventual site of ovulation (Dahm-Kähler et al. 2006).

Unbiased illustrations are difficult to create. All those who use, commission, or create visuals, as well as editors and reviewers (who supervise the use and incorporation of figures, tables, and other informative visualizations), must consider the subtle, often unintentional, messages that are conveyed. Authors must provide illustrators with information about ambiguity and bias. Artists must be aware of subliminal stereotypes and then avoid them. Reviewers and editors must carefully interrogate images for bias. Overall, awareness of possible bias and intentionality in avoiding it are necessary across the publishing process. Above, we focused on content bias; however, bias in images can also reflect cultural norms. Awareness and intentionality can help to remove bias, as the following example illustrates.

As a case study, we compare two graphics of human genital development. An older figure made without awareness of possible gender bias and a recent figure made with both awareness of potential bias and intentional action to avoid that bias. After this discussion, we provide ways to interrogate an image for potential bias.

The left graphic in Fig. 2 is an example of an illustration that has succumbed to bias. The figure is from a human embryology and developmental biology textbook (Carlson 2024; note: the same figure is used in the 4th, 2009, through 7th, 2024, editions).^8^ The graphic illustrates genital development at 7–9 weeks (top row), through 12 weeks (middle row), and ends with the late fetal stage (8–9 months, bottom row). For the bottom two rows, female anatomy is on the right, whereas male anatomy is on the left; this formatting gives males visual priority because one reads left to right in English. The graphic on the right, from a transgender public health website presents similar information. Here the presentation is reversed, giving female development priority. Since most phrasing in English is “males and females” rather than “females and males,” most images also put male information either on the left or on top. Many informational narratives also give male information priority. In fact, male-priority positioning reinforces “the sometimes overt and sometimes subtle use of illustrations, syntax, and vocabulary that makes it impossible to learn female anatomy without first learning male anatomy” (Lawrence and Bendixen 1992, 933).

The bias in the left figure goes beyond a gendered hierarchy of organization. The red ovals, which were not part of the original illustration, draw attention to more subtle areas of bias. Let’s look at the three horizontal ovals first, then the single vertical one.

In the top oval, the neutral genital tubercle has changed names from 7 to 9 weeks to become a “phallus” (a.k.a. penis), but the structure, although larger, has not otherwise changed, and other regions are not gendered. Thus, a neutral anatomical part has now become male without any concomitant change. The graphic on the right avoids the issue by providing a single neutral starting point equidistant between the two subsequent developmental paths. The newer graphic also provides more detailed and neutral labels, which are used consistently in the next stage, whereas the older graphic applies different names to 9-week vs. 12-week anatomical regions (e.g., the genital fold becomes the wall of the urethral groove in the 12-week male and is not named in the 12-week female). One positive component of the older figure is the presence of “legs” in the precursor stages to give a clearer anatomical orientation to the drawings. Such legs would only be needed once and could have been added to the newer graphic.

In the older graphic, the middle oval, encircles arrows of different length. The newer graphic avoids the necessity of arrows due to the central placement of the precursor stage. Arrows in and of themselves are not biased. However, when arrows are present, differences in their length, color, shape, or placement can result in bias. In this case, arrow length is the issue. The arrow to the male genitalia is almost three times longer than the arrow to the female genitalia, probably due to the offset placement of the male pathway from the precursor stage. Since the time frame, 9–12 weeks, is the same across sexes, the subliminal suggestion is that the female condition is closer to the infantile state. However, the developmental regions are the same size, and, in fact, the female side appears more differentiated with the addition of a new color (light purple), albeit with the loss of a label for the dark purple. The visual suggestion that female development is more infantile is reinforced by the continued presence of “legs” (lower middle oval) used in the earlier conditions. While the legs provided contextual orientation in the precursor stages, their retention in 12-week female, but not male, development subtly reinforces the message from arrow length, that is, the misconception that female physiology is more regressed than that of males.^9^

What about the newer graphic for the 10-week stage? In addition to the lack of arrows, the newer graphic employs a more neutral approach to labeling. The older graphic labeled anatomical structures separately for each pathway. The newer graphic positions the labeled key in the center between the two pathways with lines to equivalent regions across sexes. This positioning and design reinforce the similarity of the pathways, not the differences. Additionally, the space, gained from the design change, allows more extensive naming and detail.

Finally, the near-term graphics also differ. In the older graphic, the legs have been lost from the female depiction but, anomalously, the female genitalia have shrunk while the male structures have not (vertical oval). Also, the female genitalia appear to have differentiated, with new colors gained and colors lost compared with 12 weeks, while male genitalia have lost colors and structures. Overall, the inconsistent labeling and visualization in the older graphic makes comparing the developmental patterns challenging.

The “at birth” stage in the newer graphic, replicates the organization and colors present at 10 weeks. The color-coding makes obvious which regions have enlarged and which have not. While the anatomical names have become more specific to each developmental pattern, the consistent order of the labels and lines allows the reader to match the new labels with the appropriate anatomical regions. In fact, the one aberrant line (slope change) points out a major difference in the relationships between the various regions in the patterns. In contrast, the presumptive color-coding of the developmental regions in the older diagram is not as consistent as that in the newer graphic. The inconsistency leads to difficulty when trying to follow the developmental pathways of specific regions.

Overall, in the newer graphic, awareness of possible bias, and the resultant intentionality of position, color, and labeling, led to a simpler diagram that provided more information and emphasized similarity over difference. The key is awareness and intentionality. The newer graphic was from a website devoted to transgender health; the creators of the website were attuned to the nuances of gender bias and neutrality was a priority. When graphics are created with attention solely to subject matter, not audience perception, bias can result (Table 5).

**Table 5:** To avoid bias, interrogate the image. Here are questions to ask when reviewing a graphic or when designing one. They are questions to begin interrogation of visual communications. Since not all questions will be appropriate for all graphics, we give examples of cases in which bias might be present.

Source of bias	Examples of bias
Are all gametic sexes represented?	Images of meiosis that present only isogamic (i.e., male) meiosis.
Who has the priority location?	Giving males priority, for example, male to left or on top, female to right or on bottom.
Is the information the same across sexes?	Different information drawn or annotated differently for each sex (look for amount of detail, number of labeled structures, and amount of space/size).
Stereotypical use of color?	Use of pink for females, blue for males (try purple/green).
Is the terminology justified?	Use of “phallus” (see Table 2), for example, use of eponyms (Table 3) or other biased terms.
Does the content have a cultural context?	Behavioral differences that are culturally assigned primarily to a specific sex (i.e., maternal/paternal care vs. parental care).
With multiple figures in a single text, does one sex get priority?	Consistent use of the male body for all non-reproductive anatomy. With an odd number of figures, give female representation priority to balance historical bias.
Is the image misleading?	Suggesting action when none may be involved, for example, portraying ovulation as a volcanic eruption.
Does the image conflate time and space?	When presenting the stages of follicular growth as a single follicle maturing as it progresses around the ovary.

*Note:* The examples in this table often assume a binary, gametic-sex classification. Of course, the principle of even representation also applies in non-binary, multi-modal systems.

## Resources for images

Martin (2001), “The woman in the body,” illustrates early depictions of female anatomy made to appear phallus-like.

Below we list two papers that confirm androcentric bias in both web images and anatomical textbooks (Parker et al. 2017) and exclusively in web images (Guilbeault et al. 2024).

In 2017, Parker et al. analyzed 6044 gendered images from anatomy textbooks for androcentric bias. They confirmed the results of six earlier studies, which focused on text as opposed to images: males are treated as the norm and females are primarily included in sections on reproduction.

In a 2024 Nature article, Guilbeault et al. examined gender association (using ∼3500 social categories) from over 1 million, online images and billions of words from Google, Wikipedia, and Internet Movie Database. They concluded that “gender bias is consistently more prevalent in images than text.” Their analysis used social categories (e.g., jobs, professions), but one could use a similar methodology to explore text and images from online medical and reproductive physiology websites.

While we found no practical information (papers or websites) on how to reduce bias specifically in scientific illustrations, we did find a more general webpage: biases in design: hiding in plain sight in a world full of visuals by I. Persson, 26 Aug 2023, UX Collective (URL below). Persson discusses that, in design school, the “definition of what was ‘good’ or ‘universal’ had been heavily colored by a western, White, privileged social view.” The author then specifically discusses bias in typefaces, imagery, color, and symbols and provides additional resources (books, talks, podcasts, and resource lists) on these topics (https://uxdesign.cc/biases-in-design-hiding-in-plain-sight-in-a-world-full-of-visuals-6cbe64a879f2).

## Bias across borders

*[T]he specialization into large immobile gametes and small mobile gametes produced in great excess … would explain why … there is nearly always a combination of an undiscriminating eagerness in the males and a discriminating passivity in the females*.

(Bateman 1948 as cited in Tang-Martínez 2016)

Except for our section on illustrations, the biases we have presented primarily concern text in English. But such biases are present in other languages. Here we give one example using a French exploration of Bateman’s principle.

Angus Bateman’s 1948 paper, “Intra-Sexual Selection in *Drosophila*,” connects the observed sexual behaviors of fruit flies to anisogamy. Bateman suggests that the difference in energetic costs for “large immobile gametes” vs. “small mobile gametes produced in great excess” underpins sexual roles and sexual selection in nature, wherein the female is passive, like her “immobile gametes,” and the male is active, like his multitudinous “small mobile gametes.” Bateman’s principle of “anisogamy (and differential cost of gametes) as the starting point for these proposed sexual dynamics has been questioned (Dewsbury 1982; Tang-Martínez and Ryder 2005; as cited in Tang-Martínez 2016). Further, examples of sexual behavior and reproduction across taxonomy complicate the androcentric sexual roles supposedly arising from differential “cost of gametes” (Tang-Martínez 2016). For instance, the sex-role behaviors of female and male tettigoniids (bush crickets) vary depending on food availability and season, such as with *Requena verticalis*; during periods of low food availability, females compete for access to males with better nutrient spermatorphores (Tang-Martínez 2016). Further, the mating behaviors of tettigoniids in which there is a male high-energy investment (e.g., the spermatophores) complicate the binary of a female high-energy investment vs. a male low-energy investment in reproduction (Tang-Martínez 2016).

The limits of Bateman’s principle in accounting for biological underpinnings of reproductive behavior expand beyond the anglophone world. Hoquet’s (2018) text, “Cocus Naturels Ou Le Langage de La Biologie,”^10^ analyzes French examples of Bateman’s androcentric influence on reproductive research. Hoquet similarly identifies marital metaphors in writing about gamete fusion, as well as identifies economic and androcentric metaphors. “Sociobiology has made much use of the argument of anisogamy:^11^ the fact that females produce ova that are apparently much more expensive than the sperm of males. As a result, this “new science” would have us believe that women should stay at home with the children because of their large eggs” (Hoquet 2018, translated by Zoe Baker). The naturalization of human cultural sex roles is based on Bateman’s analysis of “differential cost of gametes” (Tang-Martínez 2016). To critique the androcentric logic of Bateman’s principle, Hoquet turns to another anglophone text: Martin’s (1991) analysis of American, anglophone metaphors, used to describe conception. In borrowing from Martin’s analysis of the male-centric, distorting effect that figurative speech can have, Hoquet addresses the legacy Bateman’s writing in a French context. Across linguistic and cultural contexts, the attempt to naturalize human gender roles by means of overly simplistic analyses of gamete differences hinders, rather than helps, understanding.

## Summary

The origins of cultural bias are grounded in who did the science, primarily White, Western men (Hayssen 2020). Bias is maintained by cultural acquiescence, but attentiveness to language and perspective can ameliorate the effects. We identified anthropocentrism, androcentrism, and value-laden concepts in text as well as visual imagery as forms of bias in reproductive biology. These commonly linked biases have been upheld over time through forms of communication in medical, educational, and research contexts.

The sections and tables in this paper are intended to be resources when looking for unbiased alternatives to the inaccurate terminology and historical perspectives. We acknowledge that the tables are incomplete and that not all readers will agree with our suggestions. We hope that the paradigm shift presented in this paper encourages others to identify similar areas for change in the terminology or illustration specific to their research niche, their taxon, or their native language. For instance, a paper focused on historical terminology on plant reproduction could complement Dwyer et al.’s (2022) paper on naming indigenous crops. No matter our profession (author, illustrator, proofreader, copy editor, reviewer, editor, and educator), there is much work ahead for all, as we move forward toward a more neutral framing of reproductive biology.

## Author contributions

Z.B.: conceptualization, methodology, software, formal analysis, investigation, writing—review and editing, and visualization. V.H.: conceptualization, methodology, software, investigation, writing—original draft, writing—review and editing, visualization, supervision, project administration, and funding acquisition.

## Data Availability

The data underlying Figure 1 in this article are available at https://doi.org/10.1186/1742-9994-10-66. The data were derived from a source in the public domain (Elgar et al 2013).

## Footnotes

1 Menstruation is the external discharge of the uterine endometrium.

2 Basal metabolic rate is a similar concept used in comparative physiology. BMR is easy to measure in a diverse array of captive animals but exists in wild animals only briefly, if at all.

3 Here, the authors refer specifically to non-human animals. A distinction exists between sex and gender for humans, and the language used to describe human reproductive anatomy can vary in light of this (e.g., for intersex or transgender people). For further discussions of challenging female/male binarism, gender, and human reproductive anatomy, see Sharpe et al. (2023).

4 Note that the amniotic or cleidoic “egg” is a zygote not an ovum.

5 Thanks to Joanne Benkley for coining the term “zygopositor”.

6 Eliminating “discovery” metaphors is also part of removing colonial thinking from science and is related to eliminating “firsting” in research (Max Liboiron, https://discardstudies.com/2021/01/18/firsting-in-research/).

7 Using keywords related to “Skene’s gland malignancy” (Slopnick et al. 2022).

8 The use of figures in multiple editions of textbooks is common and can maintain inherent bias over decades. Authors, reviewers, and editors should inspect figures for unintended cultural bias before repeated use.

9 An astute reviewer did not think “infantile” but instead “sexualization” of the female anatomy due to the connotation of spread legs that is not recapitulated in the male timepoint.

10 English: Natural Cuckolds, or the Language of Biology.

11 an- (a negative prefix), iso (equal), and gamy (meaning marriage).

## Notes

From the symposium “What do trade-offs mean to reproducing females?: An integrative look at whole-organism trade-offs” presented at the annual meeting of the Society for Integrative and Comparative Biology, January 2–6, 2024.
